# *Candida* isolates from pregnant women and their antifungal susceptibility in a Malaysian tertiary-care hospital

**DOI:** 10.12669/pjms.313.7072

**Published:** 2015

**Authors:** Siti Norbaya Masri, Sabariah Md Noor, Lailatul Akmar Mat Nor, Malina Osman, MM Rahman

**Affiliations:** 1 Siti Norbaya Masri, Department of Medical Microbiology & Parasitology, Faculty of Medicine & Health Sciences, Universiti Putra Malaysia, 43400 UPM, Selangor, Malaysia; 2 Sabariah Md Noor, Department of Pathology, Faculty of Medicine & Health Sciences, Universiti Putra Malaysia, 43400 UPM, Selangor, Malaysia; 3 Lailatul Akmar Mat Nor, Department of Pathology, Hospital Serdang, Selangor, Malaysia; 4 Malina Osman, Department of Medical Microbiology & Parasitology, Faculty of Medicine & Health Sciences, Universiti Putra Malaysia, 43400 UPM, Selangor, Malaysia; 5 MM Rahman, Department of Medical Microbiology & Immunology, Faculty of Medicine, Universiti Kebangsaan Malaysia, Cheras 56000, Kuala Lumpur, Malaysia

**Keywords:** Candidiasis, Pregnant women, High vaginal swab, Fluconazole, Susceptibility

## Abstract

**Objective:**

Pregnant women are susceptible to vaginal colonization and infection by yeast. The purpose of the study was to determine the prevalence of *Candida spp* in high vaginal swabs of pregnant women and their antifungal susceptibility.

**Methods:**

High vaginal swab samples received from Serdang Hospital, Selangor, Malaysia during 2011 initially had microscopic examination, Gram-staining and fungal culture. These were finally confirmed by growth in chromogenic medium (CHROMagarCandida; Difco BBL, USA) and commercial biochemical identification kit (API 20C AUX; bioMérieux, Lyon, France). Antifungal susceptibility was performed by E-test method.

**Results:**

Out of 1163 specimens 200 (17.2%) *candida spp* were confirmed from high vaginal swabs of pregnant women. *Candida albicans* (83.5%) is the most common species detected followed by *Candida glabrata* (16%) and *Candida famata* (0.05%). All *C. albicans* and *C.famata* isolates were susceptible to fluconazole while *C.glabrata* isolates were dose dependent susceptibility. First and second trimester, and diabetes were considered significant factors in patients for the vaginal candidiasis (p < 0.001).

**Conclusions:**

In pregnant women, *C. albicans* was the frequently isolated yeast from high vaginal swabs. Routine screening and treatment are important of pregnant women regardless of symptoms.

## INTRODUCTION

Although ubiquitous in nature, *Candida* species can cause various infections from superficial to invasive form. Pregnant women have a two-fold increase in the prevalence of vaginal colonization by *Candida* species compared with non-pregnant women. This association is influenced by increased levels of circulating estrogens, deposition of glycogen and other substrates in the vagina during pregnancy.^1^ Vaginal candidiasis in pregnant women was reported to cause blood stream infections particularly in low birth weight and premature infants. Using molecular typing techniques, vertical transmission of *C. albicans, C. parapsilosis*, and *C. glabrata* has been documented.^2^

In Malaysia, studies regarding the epidemiology of vaginal *Candida* infections in pregnancy are limited, with the majority being related to bloodstream infection.^3^ Fluconazole is antifungal drug effective against most of the *Candida* species, although different degrees of susceptibility among species have been described including that observed in Malaysia. The emergence of fluconazole resistance has been reported among *C. albicans*, *C. tropicalis*, and *C. parapsilosis* isolates from candidemic patients in Malaysia.^4^ Species identification & antifungal susceptibility testing of *Candida* spp from non-invasive infection is not routinely done at diagnostic microbiology laboratory in Malaysia, mainly due to limited resources and demand.

The new interpretive susceptibility criteria instituted by Clinical and Laboratory Standard Institute (CLSI^5^) that provides lower minimum inhibition concentration (MIC) break points for antifungal drugs probably change the susceptibility pattern. Hence it further justifies the need of providing local data on species distribution & antifungal susceptibility pattern of *Candida*. In this study, we determined the prevalence and risk factors associated with vaginal candidiasis in clinically symptomatic and asymptomatic cases of pregnant women in Serdang Hospital, Malaysia.

## METHODS

This cross sectional study was conducted through January 1, 2011 to June 30, 2011 at Serdang Hospital, Selangor, Malaysia. A total of 1163 high vaginal swabs (HVS) were collected from pregnant women who were attending to antenatal clinic or admitted to antenatal ward to the hospital. Patients data were obtained from hospital’s computer information system. The following data were recorded: age, diabetic status, race and age of gestation.

### Identification of Candida spp

Significant *candidal* growth was defined as presence of pure or predominant growth of *Candida spp* on fungal culture media from HVS. Isolates were identified to a species level using a chromogenic medium (CHROMagarCandida; Difco BBL, USA) as well as a commercial biochemical identification kit (API 20C AUX; bioMérieux, Lyon, France).

### Antifungal susceptibility

The in vitro activity of fluconazole was measured by the E-test (AB Biodisk, Solna, Sweden) in accordance with the manufacturer’s instructions. The MIC values were read where the inhibition elapse intersected the strip which was interpreted as the lowest concentration at which 80% of the growth was inhibited. Interpretative susceptibility criteria recommended by the Clinical and Laboratory Standard Institute (CLSI) were used to evaluate the susceptibilities of isolates.

## RESULTS

Of 1163 HVS samples 200 (17.2%) candida isolates were positive during the study period. *Candida albicans* was the most commonly isolated species, accounting for 83.5% (167/200) of the total isolates, followed by *Candida glabrata* (16%; 32/200), and *Candida famata* (0.05%; 1/200)(Table-I).

**Table-I:** Recovery of *Candida spp* from high vaginal swabs of pregnant women.

Total number of specimens	No. of positive	Species of candida	Frequency of each isolate	%
1163	200	*Candida albicans*	167	83.5
		*Candida glabrata*	32	16
		*Candida famata*	1	0.05

Factors in relation to vaginal candidiasis in pregnant women (Table-II) shows that diabetes during pregnancy and stage of gestation are significantly associated (p<0.05). We found that diabetic individual is at higher risk of having vaginal candidiasis during pregnancy compared to non-diabetic individual. In addition, first and second trimester pregnant women were the higher risk of getting vaginal candidiasis compared to the third trimester. There were no marked differences in results with respect to race and age during pregnancy.

**Table-II:** Factors related to vaginal Candidiasis in pregnant women.

Factors	Description	Candida spp.			p	Prevalence ratio
		Positive N(%)	Negative N(%)	Total		
Age Group	18-30	157(18.1%)	712(81.9%)	869	0.177	1.287
	>30	43(14.6%)	251(85.4%)	294		
Stage of Gestation	1^st^ & 2^nd^ trimester	33(27.7%)	86(72.3%)	119	0.001	0.496
	3^rd^ trimester	167(16%)	877(84%)	1044		
Diabetic Status	Diabetic	41(40.2%)	61(59.8%)	102	<0.001	2.177
	Non-Diabetic	134(15.6%)	727(84.4%)	861		
Race	Malay	157(17.3%)	751(82.7%)	908	0.873	1.031
	Non-Malay	43(16.9%)	212(83.1%)	255		

MIC ranges and MIC_50/90_ values of fluconazole against all isolates are summarized (Table-III) The MIC_50_/MIC_90_ values of fluconazole against *C.albicans* were much lower than *C.glabrata* (0.317/0.571 vs 1.516/2.728µg/mL). Our finding showed the highest MIC(6.0µg/mL) was to *C.glabrata*. However, all *Candida albicans* recovered from high vaginal swab (HVS) were susceptible to fluconazole while *C.glabrata* showed susceptible dose-dependent. None of the isolates was resistant to fluconazole. Even though there were no species-specific MIC interpretive criteria for *C.famata*, the MIC result of 0.75 µg/mL was too low; hence we conclude it as susceptible.

**Table-III:** MIC values and ranges (µg/mL) of fluconazole against the isolates of *Candida spp*.

*Candida* species (n)	Antifungal Agent	Mean MIC_50_(µg/mL)	Mean MIC_90_ (µg/mL)	MIC range (µg/mL)
*C. albicans* (167)	Fluconazole	S = 0.317	S = 0.571	0.094 – 2.000
*C. glabrata* (32)	Fluconazole	S-DD = 1.516	S-DD = 2.728	1.000 – 6.000
*C. famata* (1)	Fluconazole	ND	ND	0.75

S = susceptible, S-DD = susceptible dose-dependent.

ND = Not done (the number of isolates was too small for full analysis)

## DISCUSSION

In the present study, the recovery rate of *Candida spp* from pregnant women was 17.2% with *C.albicans* being the most common species detected (83.5%), followed by *C. glabrata* (16%). Similarities were observed with previous reports on vaginal candida spp distribution in Malaysia where *C. albicans* and *C. glabrata* were found 70% and 15% respectively^3^ This finding is also consistent with reports by investigators from other countries, in which *C.albicans* and *C.glabrata* were the most common *Candida* species isolated in vaginal candidiasis.^6^ A study in Turkey reported that clinically and mycologically confirmed cases of vulvovaginal candidiasis were detected in 139 (37.4%) of 372 pregnant women.^7^ In one study, a higher prevalence of vaginal candidiasis in pregnant women where 53% of patients with clinical diagnostic candidiasis had culture-positive vaginal isolates.^6^ Our prevalence rate was relatively low, similar to that found in other studies.

There is an evidence that eradication of *Candida* in pregnancy may reduce the risk of spontaneous preterm birth who were treated for vaginal candidiasis^8^ though a study reported that vaginal candidiasis in pregnancy was not associated with preterm birth.^9^

A striking observation in this study is that non-albicans *Candida* (NAC) species was very much lower than *C.albicans* in vaginal candidiasis (16.5% vs 83.5%). In a series of 50 cases of candidemia in Malaysia showed that NAC species constituted 60% of all yeast isolates and the most prevalent NAC species was *C.tropicalis*.^10^ Similarly, in another report 55.3% of the isolates collected from patients with invasive candidiasis were NAC species with *C.parapsilosis* being the most common one.^4^ The investigators also observed that though *C.albicans* was the predominant species in pregnant women, but in case of immunocompromised patients 82% of isolates were non-albicans *Candida* species. It was observed that C.albicans was the predominant species for vaginal candidiasis while *C. parapsilosis* was the most prevalent organism isolated from blood cultures.^11^ The shift in distribution of candida species particularly in invasive infection may be associated with the widespread use of prophylactic or empirical antifungal therapy and the increase in number of immunocompromised hosts.

Majority of studies have reported that incidence of candidiasis increases with gestational age.^12^ In contrast, our study showed the risk of getting vaginal candidiasis is higher during the 1^st^-and 2^nd^ trimester as compared to 3^rd^ trimester. Parveen *et al*.^13^ reported that there was no significant association between vaginal candidiasis and trimester of pregnancy.

In the light of previous studies our result showed a significant association between diabetes and vaginal candidiasis in which of 143 diabetic patients, 41 had vaginal candidiasis during pregnancy (p<0.001). In contrast, other authors noted that diabetes, or impaired glucose tolerance during pregnancy was not associated with vaginal candidiasis.^14^ In a study it was observed that women with diabetes experienced significantly higher isolation rate of both *C.albicans* and non-albicans *Candida* compared to non-diabetics.^15^ In addition to diabetes, the susceptibility to candidiasis in pregnant women is much higher due to hormonal changes during pregnancy.

There is an evidence to suggest that screening for and eradication of candida during pregnancy may reduce the risk of preterm delivery. In a large Austrian randomized controlled trial, spontaneous preterm birth occurred in 8/289 women treated for candidiasis versus 22/291 women with candidiasis in the control group (OR 0.35, 95% CI 0.14-0.84 P=0.009).^16^

In this study, all *C.albicans* isolates were susceptible to fluconazole. Study conducted in United States and Brazil were also found that all *C. albicans* vaginal isolates were susceptible to fluconazole.^17^ However, although a report showed that 13.5% of isolates were resistant to fluconazole in *Candida albicans* vulvovaginitis.^18^ According to CLSI^5^ interpretive guideline, there is no susceptible MIC breakpoint for fluconazole against *C.glabrata* and MIC of <32µg/mL is interpreted as susceptible dependent upon dose (S-DD), to indicate that dosage escalation may be required adequately to treat infections caused by isolates with a higher MICs. In this study, *C.glabrata* isolates showed MIC range 1.00 µg/mL – 6.00µg/mLand 90% of them had MIC ≤ 2.728µg/mL which is much lower than 32µg/mL. This is in contrast to a study in Taiwan which reported a high fluconazole-resistant rate (64%) among *C.glabrata* from vaginal isolates.^6^

Our study showed that *Candida glabrata* had higher MIC_50&90_ than *C. albicans*. *Candida glabrata* is associated with drug resistance due to either over-expression of membrane ergosterol synthesis enzyme or the strains genetic modification.^19^ Inappropriate use of antifungal agents has led to the emergence of antifungal resistance.^7^ In the present study, although fluconazole showed good activity against all our isolates, the pattern could be changed with time as seen in other countries. Therefore, monitoring the activity of antifungal drugs is of concern particularly among pregnant women as the choice of treatment is limited by their side effects.
